# Extracting Interactions between Flying Bat Pairs Using Model-Free Methods

**DOI:** 10.3390/e21010042

**Published:** 2019-01-09

**Authors:** Subhradeep Roy, Kayla Howes, Rolf Müller, Sachit Butail, Nicole Abaid

**Affiliations:** 1 Physical Computing Lab., Virginia Tech, Blacksburg, VA 24061, USA; 2 Department of Biomedical Engineering and Mechanics, Virginia Tech, Blacksburg, VA 24061, USA; 3 Department of Mechanical Engineering, Virginia Tech, Blacksburg, VA 24061, USA; 4 Shandong University-Virginia Tech International Laboratory, Shandong University, Jinan 250100, China; 5 Department of Mechanical Engineering, Northern Illinois University, DeKalb, IL 60115, USA

**Keywords:** bat interaction, convergent cross map, curvature, transfer entropy

## Abstract

Social animals exhibit collective behavior whereby they negotiate to reach an agreement, such as the coordination of group motion. Bats are unique among most social animals, since they use active sensory echolocation by emitting ultrasonic waves and sensing echoes to navigate. Bats’ use of active sensing may result in acoustic interference from peers, driving different behavior when they fly together rather than alone. The present study explores quantitative methods that can be used to understand whether bats flying in pairs move independently of each other or interact. The study used field data from bats in flight and is based on the assumption that interactions between two bats are evidenced in their flight patterns. To quantify pairwise interaction, we defined the strength of coupling using model-free methods from dynamical systems and information theory. We used a control condition to eliminate similarities in flight path due to environmental geometry. Our research question is whether these data-driven methods identify directed coupling between bats from their flight paths and, if so, whether the results are consistent between methods. Results demonstrate evidence of information exchange between flying bat pairs, and, in particular, we find significant evidence of rear-to-front coupling in bats’ turning behavior when they fly in the absence of obstacles.

## 1. Introduction

Animal groups, such as bird flocks, fish schools, and insect swarms, frequently exhibit coordination—often referred to as collective behavior—that results from social interactions among individuals [1]. In nature, social animals take advantage of collective behavior to locate food sources, avoid predators, and find mates [1]. Interaction among individuals in a group is a strong determinant of collective behavior, whereby information propagates across the group and enables high-level coordination. Observing these interactions from the perspective of an individual is generally not possible, so the evidence of interaction through the resulting behavior is typically studied. However, when the type of behavior resulting from interaction is not known, a model-free approach that uses data from laboratory or field experiments is needed. Since interactions and information exchange can be either unidirectional or bidirectional, indicating one- or two-way influence, this distinction must also be quantified.

In the present study, we focus on organisms that use active sensory systems [2,3]. Animals that use passive sensing receive signals from the environment, whereas animals that use active sensing generate a signal that interacts with the environment and is then received. A fitting example of active-sensing animals is bats that echolocate with ultrasound. These animals can vary the intensity, direction, and timing of their signal and thus have a high level of control that enables the extraction of targeted information from their environment [4]. Moreover, it has been postulated that bats flying in groups also intercept the echolocation signals of their peers [5,6], particularly those that live in high-density groups. To directly observe the information that a bat may gather from the sensing signals of its peers, acoustic data need to be captured from the bat’s location via an onboard microphone, which is a difficult problem due to the small size and low weight of these animals. In spite of these challenges, it is important to understand how bats interact via active sensing, since it may have advantages over passive sensing for groups of individuals. Particularly, active sensing promotes information sharing whereby one individual bat may eavesdrop and use the reflected signal from its peer (passive listening) to sense obstacles or detect a food source [6]. Furthermore, active sensing allows bats to potentially send information directionally in space—for example, to purposefully confuse conspecifics during competitive hunting [7]—whereas vision or passive sensing do not. In this study, we hypothesize that the bats flying in pairs influence their peers’ motion, which we define as leadership. This definition of leadership is consistent with previous studies, such as in [3] and [8], where leaders are identified by the measure of their influence on their group’s motion.

To quantitatively assess navigational leadership roles in bat swarms, considering their unique sensing capabilities, we explore model-free methods that can be used to extract interactions from flying bat pairs and detect leadership using flight trajectory data. Specifically, we focus on trajectories of pairs of bats wherein one bat is positioned in the front and the other in the rear, and we identify the dominant direction (front-to-rear or rear-to-front) of coupling using these methods. A recent study investigated the role of multiple sensing cues in pairs of flying bats—namely, echolocation and vision—and revealed the subsequent emergence of leader–follower relationships [3] in terms of bats’ heading direction when flying near an obstacle. We explore the emergence of leadership in bat pairs flying both with and without obstacles, our motivation being to identify the spatial position (front/rear) occupied by leaders.

A classical approach to identify leader–follower interactions is to compute a cross-correlation between two time series, which gives a maximum correlation value at some nonzero lag; the leading time series is identified as leader and the lagged time series as follower. Cross-correlation has been applied to detect leadership in animal group motion; for example, it was used to find hierarchy in pigeon flocks [8]. However, a major limitation of cross-correlation is that it assumes a linear dependence and is sensitive to noise which is typical in real-world systems. Alternative methods are model-free approaches that do not assume behaviors a priori and are capable of identifying nonlinear relationships, unlike cross-correlation which assumes a linear model to fit the dataset. Increasingly popular among these methods are transfer entropy (TE) [9] and convergent cross map (CCM) [10]. Transfer entropy is an information-theoretic approach and measures information exchange between two or more random variables, whereas convergent cross map is grounded in dynamical systems theory and has the potential to detect a dominant direction of coupling between two time series. Transfer entropy has found applications in different fields, including neuroscience [11], finance [12], communications [13], and climate science [14], whereas CCM has been successfully applied in ecology [10], neuroscience [15], and marketing [16]. Recently, information-theoretic approaches and particularly transfer entropy have received increasing interest for studying interactions in animal group motion, such as in fish [17,18,19], crabs [20], meerkats [21], and insect swarms [22]. On the contrary, in spite of being qualified as a potential framework to detect directed coupling, CCM has not previously been used in the context of animal groups.

In this study, we compared two model-free methods for measuring influence within a pair of individuals by quantifying directed coupling between their motions. This study used field data from bats in flight to explore these methods. We selected bats since their active sensing makes them a unique example of potential directional pairwise interaction. To differentiate the bats’ pairwise interaction from background similarities in flight trajectories, we defined a control condition to account for information that bats may receive from the geometry of the environment. This study is based on the assumption that interactions between pairs of bats are evidenced in their flight path patterns. With results from the control condition, we tested the hypotheses that directed coupling can be significantly detected for pairs of bats flying together and that this coupling depends on their behavior with respect to obstacles introduced in their environment. We further studied consistency between the different methods.

## 2. Materials and Methods

### 2.1. Methods to Detect Directed Coupling

We used two model-free methods, TE and CCM, to identify interactions in pairs of bats flying together. These methods are capable of quantifying the strength of coupling and hence can detect the dominant coupling direction, which is then inferred as the causal variable. Here, we briefly explain how these methods work; an example with toy data is provided in Appendix A for demonstration.

#### 2.1.1. Transfer Entropy

Schreiber [23] formalized the idea of transfer entropy to measure information flow between two time series based on Shannon entropy. Shannon entropy is a measure of the expected amount of information in a time series, defined as

(1) $$H\left(Y\right)=-\sum_{y\in Y}\mathrm{Pr}\left[y\right]\log_{2}\mathrm{Pr}\left[y\right],$$

where Pr[y] is the probability density function for a random variable *Y* taking the value *y*, and Y refers to the set containing all possible realizations of *Y*. Transfer entropy extends this concept to compare the entropy in one time series given some knowledge of another time series; information is said to flow between time series if the entropy of one decreases by conditioning the other. TE from *X* to *Y*, $T_{X\to Y}$, computes information transfer from *X* to *Y* by measuring the degree of uncertainty resolved to predict the future of *Y* from its present, using the additional knowledge of *X* at present. Formally, this is

(2) $$T_{X\to Y}=\sum_{\begin{array}{c} y(t+1)\in Y(t+1),\\ y(t)\in Y(t),\\ x(t)\in X(t) \end{array}}\mathrm{Pr}\left[y\left(t+1\right),y\left(t\right),x\left(t\right)\right]\log\frac{\mathrm{Pr}[y(t+1)|y(t),x(t)]}{\mathrm{Pr}[y(t+1)|y(t)]},$$

where Pr[y(t+1)|y(t)] and Pr[y(t+1)|y(t),x(t)] denote the probability of y(t+1) conditioned on only y(t) and both y(t) and x(t), respectively; Pr[y(t+1),y(t),x(t)] denotes joint probability. Depending upon the logarithm base selected, the unit of TE can either be bits (base 2) or nats (base *e*). In the absence of influence of *X* on *Y*, Pr[y(t+1)|y(t),x(t)]=Pr[y(t+1)|y(t)], and hence, $T_{X\to Y}$ equals zero. By construction, transfer entropy is an asymmetric quantity so that $T_{X\to Y}$ is not equal to $T_{Y\to X}$, and the idea is used to identify the dominant direction of information flow or coupling. Transfer entropy can also be expressed in terms of conditional entropy as $T_{X\to Y}=H\left(Y\left(t+1\right)|Y\left(t\right)\right)-H\left(Y\left(t+1\right)|Y\left(t\right),X\left(t\right)\right),$ where H(Y) is the Shannon entropy above. The probability distribution function (PDF) used in these definitions can be calculated by using different techniques [24]: for example, partitioning each time series variable into a finite number of discrete bins and then computing the relative frequency of occupancy of the samples in the bin (binning estimators); using a kernel function that measures similarity between pairs of samples given resolution or kernel width *r* (kernel-estimators); or the recent Kraskov, Stogbauer, and Grassberger (KSG) method which uses a dynamically altered kernel width in terms of *K* nearest-neighbors ($k_{\mathrm{nn}}$). We performed TE analysis with the Java Information Dynamics Toolkit (JIDT) for Matlab [25], which is an open-source code to implement various information-theoretic measures from time series data. In all analyses, unless otherwise noted, we used the KSG method to compute TE since it has been shown to decrease errors in PDF estimation [26], and the unit is in nats.

#### 2.1.2. Convergent Cross Map

CCM was first introduced in [10] and is based on an algorithm that compares the ability of lagged components of one time series variable to estimate the dynamics of another. Given two time series X(t) and Y(t), where *t* denotes the time index and, in the case of unidirectional coupling, (X→Y), the dominant variable (X) leaves signatures on the affected variable (Y). Hence, the past values of *Y* can better estimate the values of *X* at the corresponding times. This idea is implemented by constructing a shadow manifold from time-delayed projections of each time series independently. A more detailed description of the algorithm can be found in the supplementary materials of [10] and in [27] which can be summarized as follows. We first constructed an embedding for the time series *X* by defining *E*-dimensional points of the form (X(t),X(t−τ),X(t−2τ),⋯,X(t−(E−1)τ)), where τ is a constant time delay. These data points are defined as t∈{1+(E−1)τ,2+(E−1)τ,⋯,L}, where *L* is the length of the time series used for the embedding, called the library size. The *E*-dimensional manifold containing these points is called the shadow manifold of *X*, or $M_{X}$. Similarly, a shadow manifold of *Y*, called $M_{Y}$, was constructed independently. Next, a cross-mapped estimate of Y(t) was obtained by locating the nearest neighbors on $M_{X}$. Finally, cross map skill was evaluated as Pearson’s correlation coefficient between *Y* and estimated *Y*, where correlation coefficients close to one indicate better estimation. The negative correlation values were converted to zeros and were treated as insignificant, indicating that the CCM estimate lacked information about the dynamics of the other variable. Hence, the CCM values range between 0 and 1 and are unitless. The cross map estimation improves with increased library size, L, and converges to a constant value asymptotically with *L*. To see this convergence, CCM computes the cross map skill over all possible sequential segments of data having the length *L* from the given time series, which are then averaged. For example, let us assume we have a time series of length 100. To compute the CCM value at L=10, we select all possible sequences of length 10, i.e., {1⋯10},{2⋯11},⋯,{91⋯100}, and then compute the average of the cross map skill over these sequences. The values of *L* are provided in increments as input, and the maximum of *L* is equal to the length of the given time series data. As *L* increases, the *E*-dimensional manifold gets dense, resulting in reduced estimation error and convergence. CCM identifies the dominant variable by comparing the asymptotic value of cross map skill between the two directed computations. In real-world datasets, due to the presence of observational error, process noise, and lack of enough data, the asymptotic cross map skill may not converge to one [10]. Also, it needs to be noted if more than two variables are causally related in a system; a pairwise analysis needs to performed to detect all such links, and, in such scenarios, the value of convergence may not reach one. Please refer to the five-species model example in [10] for more details. In such a scenario, the CCM value that is closer to one is interpreted as the dominant coupling direction [10]. CCM in the present study was implemented using the rEDM package [28] in R, where the time delay (τ) used for construction of the shadow manifold was set to 1 by default, and the optimal embedding dimension *E* was evaluated following the Simplex Projection method [29].

#### 2.1.3. Differences between TE and CCM

**Figure 1:**
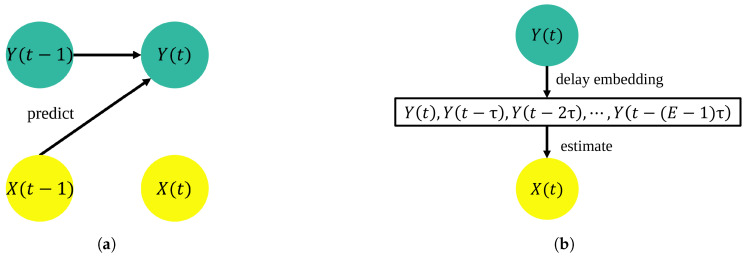
Visualization of transfer entropy (TE) and convergent cross map (CCM) using a schematic representation of when *X* (cause) drives *Y* (effect). (**a**) TE’s underlying assumption is that past values of the effect, along with the additional knowledge of past values of the cause, result in improved prediction of the future of the effect (denoted by black arrows). (**b**) CCM’s underlying assumption is that the cause leaves imprints on the effect, and hence, the effect is a better predictor for estimating contemporaneous values of the cause (denoted by black arrows), compared with using the cause as a predictor for the effect. CCM uses a delay-embedding technique.

Figure 1 explains the underlying assumptions on which TE and CCM rely to detect directed coupling between two time series. Particularly, the assumption of TE is that the cause precedes the effect, and hence, the cause can help in forecasting the effect, whereas the assumption of CCM is that the cause leaves imprints on the effect, and hence, the effect can better estimate the cause. Apart from the difference in these methods’ underlying assumptions, TE uses probability distributions of time series data that eliminate all dynamic information beyond comparing pairs of successive time steps, whereas CCM retains dynamics with a time-delay embedding. Moreover, CCM utilizes the idea of convergence with increased library size/time series length. On the contrary, TE simultaneously uses the entire dataset to measure information transfer. Furthermore, CCM is a method developed to directly identify causality, whereas TE measures differences in information transfer that can be used to detect causality. Hence, these two model-free methods provide qualitatively different indicators of pairwise interactions. Both the approaches are able to correctly identify the directed coupling for a toy dataset (see Appendix A). The present study compares these two methods when applied to a real-world dataset of bats’ flight, with TE used to find evidence of pairwise interaction and CCM used to detect leadership. It is important to note that TE does not directly infer causality: in other words, leadership. We can indirectly access leadership using TE by identifying the level of asymmetry in TE values in either direction of two time series variables. However, CCM is developed to identify the cause–effect relationship: in other words, the leader–follower relationship.

Table 1 summarizes the input parameters and the outputs for both TE and CCM.

### 2.2. Experimental Setup and Data Collection

**Figure 2:**
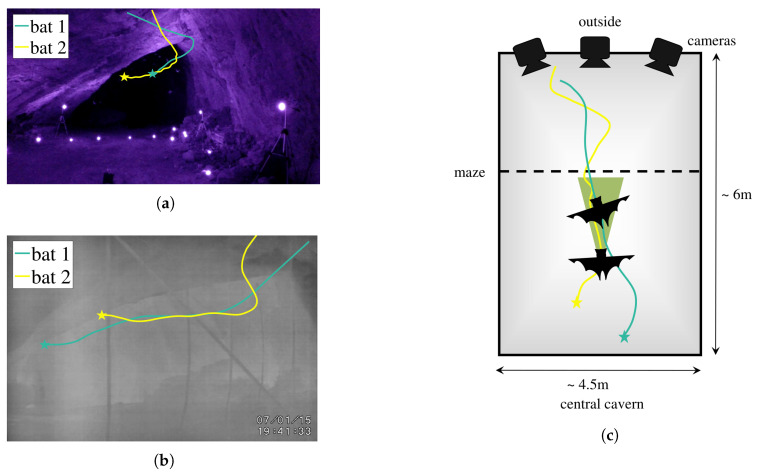
Visualization of experiments with two bats flying as a pair. Two different experimental setups were used for filming the videos of the bats’ flights (**a**) GoPro cameras with infrared illumination in 2014 and (**b**) thermal cameras in 2015. (**c**) A top-view schematic of the capture volume for the 2015 setup demonstrates a pair of bats flying in the presence of obstacles or a “maze”. The green triangle demonstrates how sonar sensing can result in directed information transfer from bats that are spatially behind their partners. In all subfigures, the star symbol is used to represent the start of the path.

For this study, we used flight trajectory data from wild bats filmed in two field experiments in the summers of 2014 and 2015 at a cave on Mount Lian Tai in Shandong Province, China. This cave served as a roost to a colony of approximately 2000 mouse-eared bats (*Myotis* sp.). In 2014, the bat trajectories were filmed in the absence of any obstacles. In 2015, we introduced obstacles to the bats’ flight paths by arranging thin films in a vertical maze to compel the pairs of bats to coordinate in the presence of obstacles. Figure 2 demonstrates the two different experimental setups and a schematic of two interacting bats as they fly together in the presence of obstacles. In 2014, six GoPro cameras (video acquisition rate 60 Hz) with infrared filters [30] were used to film the bats’ flights; in 2015, three thermal cameras (video acquisition rate 25 Hz) were used. This experiment was approved by the Virginia Tech Institutional Animal Care and Use Committee (protocol 14-123) and was carried out in accordance with the American Society of Mammalogists guidelines [31].

A total of 30 pairs of bats (10 pairs from 2014, 20 pairs from 2015) were tracked as they flew through the capture volume, the 3D volume within which bat trajectories were filmed. The capture volume was an approximately 6 m long cross-section of a cave entrance which bats navigated to exit the cave after sunset. The 3D coordinates of bat trajectories from the raw video data were extracted from multiple camera views of the capture volume using a custom Matlab script and proprietary and open-source calibration scripts [32,33]. Bat pairs were selected for analysis if two bats flew together with no other bats in the tracking volume. We refer to bat 1 and bat 2 as the bats that entered the capture volume first and second, respectively. We fitted a third-order spline through the 3D coordinates of bat trajectories and resampled at 180 Hz. Further details regarding the choice of sample rate are provided in Appendix B. The median values of the trajectory lengths traversed by bat 1 and bat 2 for all 30 pairs are 3.54 m and 2.59 m, respectively, and the median duration of flight is 1.26 s.

Trajectory curvature was used as the 1D time series for this analysis since we considered steering, or change in direction, to measure interaction. This quantity has been previously used to study animal behavior, such as laboratory insects [34]. To compute curvature, we differentiated the spline to generate 3D velocity and acceleration over time. Curvature values at each time step were calculated using the following equations:

(3) $$\begin{array}{ccc} a_{n} & =a-a_{t}=a-\frac{a\cdot v}{{\left||v|\right|}^{2}}v\;\;\mathrm{and}\;\rho & =\frac{\left|\right|a_{n}\left|\right|}{{\left||v|\right|}^{2}}, \end{array}$$

where a, $a_{n},$ $a_{t}$ are acceleration, normal acceleration, and tangential acceleration vectors, respectively; *v* is the velocity vector; and ρ is curvature. To verify the sensitivity of the results to the choice of independent variable, we additionally used the 3D velocity data as input for the multivariate functionality of transfer entropy. Further details regarding the data processing, trajectory length, and duration of flight corresponding to each pair are provided in Table A1 of Appendix B.

Considering data from the two experiments, we categorized the bat pair trajectories into three groups based on flight behaviors as follows. The first 10 bat pairs, which were from experiments conducted in 2014, flew in the absence of any introduced obstacles or maze and compose the group referred to as “No-Maze” (NM). The remaining bat pairs from experiments in 2015 flew in the presence of an obstacle maze and were further subdivided into two categories based on whether the bat pair traversed the maze or avoided it by turning. Accordingly, we categorized the 20 pairs of trajectories in the 2015 dataset into the 14 pairs that traversed the maze, called “Maze Cross”(MC), and the 6 pairs that avoided the maze by turning, called “Maze Turn”(MT).

#### Data Availability

The datasets generated and analyzed for the current study are available from the University Libraries, Virginia Tech repository, https://data.lib.vt.edu/collections/g732d9021 (doi:10.7294/W4639MWR).

### 2.3. Data Analysis

For each bat pair, bat 1 entered the capture volume first and bat 2 followed. Hence, there was a portion at the beginning and end of the time series when one of the bats flew by itself in the capture volume. In order to perform TE and CCM, we used only the portion of the time series data when both bats were in the capture volume.

TE and CCM were implemented on all 30 bat pairs and on the data partitioned into three subgroups by behavior. The ensemble data for all 30 bat pairs is referred to as “All”. To interrogate the effect of behavior, we created ensemble datasets similarly from subgroups that exhibited NM, MC, and MT behaviors. For each implementation, we created an ensemble dataset by concatenating the curvature time series for bat 1 and bat 2 from multiple pairs independently. We chose to concatenate the time series data for the analysis, which is consistent with the previous study in [35]. Concatenation is conducive to obtaining the correct estimation of PDFs for TE, and the larger dataset fills the attractor manifold more densely, thus promoting accuracy in cross map estimates for CCM. An alternate approach is to perform the analysis separately for each pair; however, the dataset may be too small since the methods used in our study are data hungry. For the analysis in this paper, the total number of time steps, rather than the number of bat pairs, determines the number of the sample size.

To create a baseline for values of TE and CCM that do not denote interaction, we defined a control condition by shuffling data from the entire dataset and from each subgroup. Specifically, we manufactured 100 randomly shuffled datasets within which partners were scrambled. That is to say, each bat 1 was compared with a bat 2, but not a bat with which it coincided in time during the experiment. When we matched time series of different lengths, we selected the time series corresponding to shorter library size and truncated the end of the larger one to make it of the same size. Ensemble time series from actual bat pairs and shuffled pairs were normalized to a zero mean and unit variance before implementing CCM and TE, consistent with previous analyses [10,36]. After this analysis, we compared our experimental results to these control conditions by computing the 95% confidence interval based on the 100 randomly shuffled datasets. We consider the experimental result to be significant if the value is higher than the 95% confidence interval [37] and insignificant if it is lower. The main idea is to distinguish a pairwise interaction beyond the effect of external factors, such as the geometry of the cave itself; this analysis tests the hypothesis that the pairwise interaction requires information exchange that is additional to the similarities in trajectories due to the fact that all the bats flew in the same physical space.

## 3. Results

**Figure 3:**
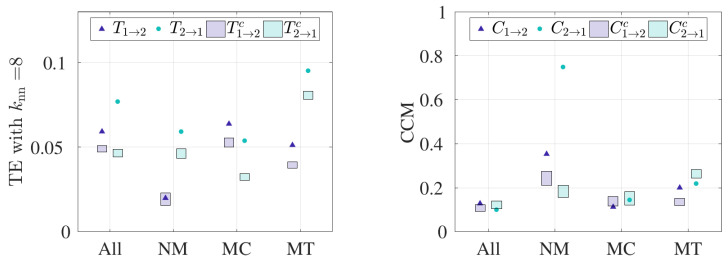
Summary of the results. Results from computing (**a**) TE and (**b**) CCM on ensemble curvature time series data. Control conditions are shown as the 95% confidence interval in the shaded regions. TE and CCM were implemented on ensembled time series data for all bat pairs (total of 30 pairs), “No-Maze” (NM) pairs (total of 10 pairs), “Maze Cross” (MC) pairs (total of 14 pairs), and “Maze Turn” (MT) pairs (total of 6 pairs). We used $k_{\mathrm{nn}}=8$ for the TE measurement and the CCM value at the maximum library size available. High values of TE imply high information transfer, and low values of CCM imply lack of leadership.

Results from computing the TE and CCM for all pairs and for the three subgroups categorized by behavior are shown in Figure 3. For the TE measurement, we fixed $k_{\mathrm{nn}}=8$ so that the resolution width in estimating transfer entropy is neither too large nor too small [26]; sensitivity to this parameter is discussed below. Alternatively, CCM relies on the asymptotic value of cross map skill to infer directed coupling, so we used the maximum library size available for each dataset. We denote transfer entropy (respectively CCM) from bat 1 to bat 2 and from bat 2 to bat 1 as $T_{1\to 2}$ (respectively $C_{1\to 2}$) and $T_{2\to 1}$ (respectively $C_{2\to 1}$); for the control condition, the values are denoted as $T_{1\to 2}^{c}$ (respectively $C_{1\to 2}^{c}$) and $T_{2\to 1}^{c}$ (respectively $C_{2\to 1}^{c}$). The shaded regions refer the to 95% confidence interval of the control condition for bat 1 and bat 2.

For all bats, TE demonstrates bidirectional coupling between bats 1 and 2 (Figure 3a), as significance above the control condition is observed in either direction. However, statistical significance is not reached when CCM was implemented on all pairs, as seen in Figure 3b.

Analysis by behavior reveals significant results with respect to the control condition when NM pairs are considered. For NM pairs, TE recognizes unidirectional coupling from bat 2 to 1, with statistical insignificance established in the opposite direction. Interestingly, CCM detects significant results in either direction for NM pairs. However, $C_{2\to 1}$ (∼0.75) is much larger than $C_{1\to 2}$ (∼0.35); thus, CCM recognizes bat 2 to bat 1 as the dominant coupling direction, which is consistent with TE results. In other words, both TE and CCM identify bat 2 as the leader when bat pairs fly in the absence of externally provided obstacles. When bat pairs in the presence of obstacles (MC and MT) were considered, TE reveals bidirectional coupling in each case, as observed in Figure 3a. However, CCM does not indicate the presence of directed coupling for MC pairs, whereas, for MT pairs, a significance is observed in $C_{1\to 2}$ (Figure 3b). However, since the value of $C_{1\to 2}$ is less than $C_{2\to 1}$, we cannot conclude that bat 1 is the leader.

**Figure 4:**
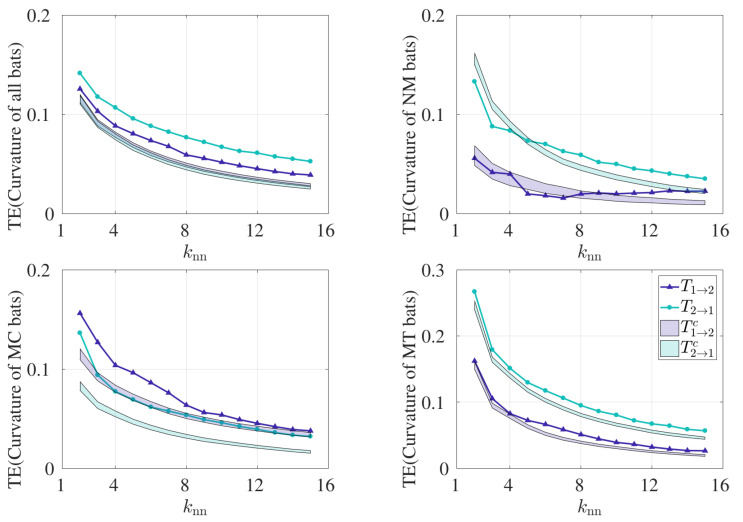
Transfer entropy calculated using curvature time series. $T_{1\to 2}$ and $T_{2\to 1}$ are calculated for varying values for $k_{\mathrm{nn}}$. Control conditions $T_{1\to 2}^{c}$ and $T_{2\to 1}^{c}$ are shown as the 95% confidence interval in the shaded regions. TE was implemented on ensembled time series data for (**a**) all bat pairs (30 pairs combined, $L_{\max}=9514$), (**b**) NM pairs (10 pairs combined, $L_{\max}=1317$), (**c**) MC pairs (14 pairs combined, $L_{\max}=3452$), and (**d**) MT pairs (6 pairs combined, $L_{\max}=4745$), where $L_{\max}$ denotes the maximum library size in each category.

In order to understand the sensitivity of the TE results to the parameter choice used in this analysis, we varied $k_{\mathrm{nn}}$ from 2 to 15, as shown in Figure 4. We anticipated that statistical significance may not be obtained for large and small values of $k_{\mathrm{nn}}$, which are known to adversely affect the estimation of PDFs. Figure 4a demonstrates bidirectional coupling between bats 1 and 2 for all bats, and significant results are observed in either direction for the entire range of $k_{\mathrm{nn}}$ values. Hence, the results for all pairs are insensitive to the resolution width used in the estimation of TE. Splitting by behavior, we observe that the TE results are sensitive to the choice of $k_{\mathrm{nn}}$ values, as shown in Figure 4b. Specifically, considering NM pairs, significant results are observed in $T_{2\to 1}$ for $k_{\mathrm{nn}}$ values greater than 5, as evidenced from Figure 4b, and $T_{1\to 2}$ loses significance at higher values of $k_{\mathrm{nn}}$ in MC pairs. However, from Figure 4c,d, we find significance in the transfer entropy results from bat 2 to 1 ($T_{2\to 1}$) across all choices of $k_{\mathrm{nn}}$ values. Hence, we fixed $k_{\mathrm{nn}}$ equal to 8 for the comparison between methods, where $k_{\mathrm{nn}}$ is neither too large nor too small to result in degenerate PDF estimation.

**Figure 5:**
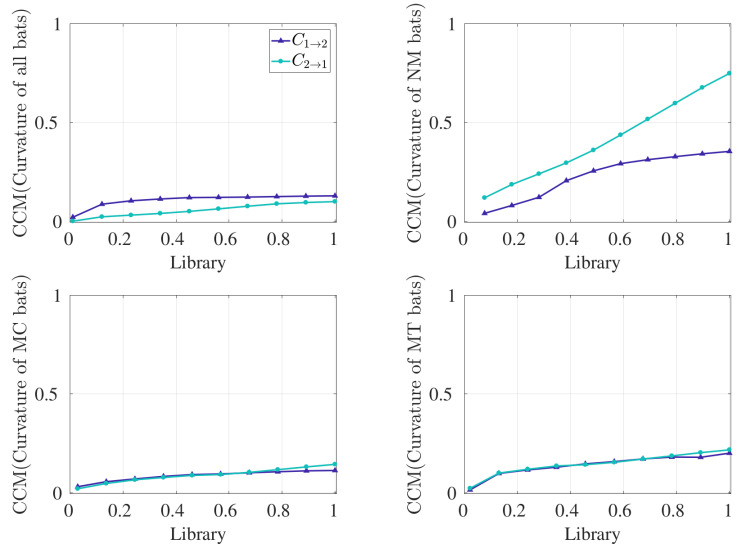
Convergent cross map skill calculated using curvature time series. $C_{1\to 2}$ and $C_{2\to 1}$ were calculated for increasing library sizes. CCM was implemented on ensembled time series data for (**a**) all bat pairs (30 pairs combined, $L_{\max}=9514$), (**b**) NM pairs (10 pairs combined, $L_{\max}=1317$), (**c**) MC pairs (14 pairs combined, $L_{\max}=3452$), and (**d**) MT pairs (6 pairs combined, $L_{\max}=4745$).

For CCM, it should be noted that the estimation of significance at intermediate library sizes is not conclusive in terms of identifying directed coupling, as CCM relies on the converged value of cross map skill. We performed CCM on experimental data at different library sizes, shown in Figure 5, to demonstrate how the cross map skills increase with increasing library size and can be used to verify convergence. We note from Figure 5b that CCM from bat 2 to bat 1 increases to a high but non-constant value in NM pairs with increasing library size, reflecting strong directed coupling. However, we can observe that the length of the time series for the NM pairs is not long enough for $C_{2\to 1}$ to attain convergence.

## 4. Discussion

**Figure 6:**
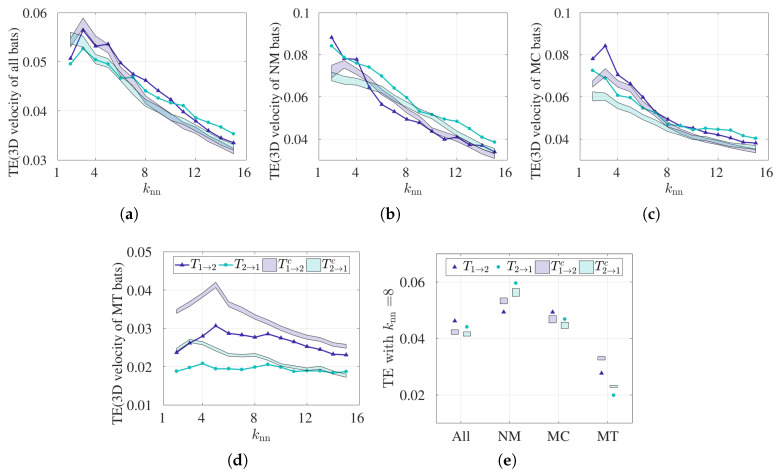
Transfer entropy calculated using 3D velocity time series. $T_{1\to 2}$ and $T_{2\to 1}$ were calculated for varying values for $k_{\mathrm{nn}}$. Control conditions $T_{1\to 2}^{c}$ and $T_{2\to 1}^{c}$ are shown as the 95% confidence interval in the shaded regions. TE was implemented on ensembled time series data for (**a**) all bat pairs (30 pairs combined, $L_{\max}=9514$), (**b**) NM pairs (10 pairs combined, $L_{\max}=1317$), (**c**) MC pairs (14 pairs combined, $L_{\max}=3452$), and (**d**) MT pairs (6 pairs combined, $L_{\max}=4745$), where $L_{\max}$ denotes maximum library size in each category. (**e**) Summary of the TE results for $k_{\mathrm{nn}}=8.$

- **Statistically significant coupling evidences pairwise interaction.**
  TE and CCM results support the hypothesis that pairwise interactions involve additional coupling above control similarities in trajectories. TE captures significant bidirectional coupling between bats in all, MC, and MT pairs and significant unidirectional coupling from the rear to the front bat in NM pairs. On the other hand, CCM identifies leadership in the rear bat for NM pairs, and it gives no indication of leadership in the other pairs. Therefore, all unidirectional results are consistent, and bidirectional information flow (from TE) is equivalent to a lack of a directed causal relationship (from CCM). Since we selected the curvature time series as input for both analyses, these results evidence a relationship between how bats turn as they fly together. In this context, bidirectional coupling means that the two paired bats’ turning behaviors are mutually linked, while unidirectional coupling means that one bat drives the other to turn or not. In contrast, if we were to use velocity data for another TE analysis, then we would be able to test how the bats’ headings or speeds related.
  It is important to note that an alternative control condition can be used whereby the manufactured data can be shuffled in time. Shuffling in time refers to arbitrarily reordering the data between time steps for a given series, thus distorting its sequence. However, such a control is very liberal and leads to a zero mean and a standard deviation close to zero for virtually every case, resulting in statistical significance for all cases of TE and CCM. We chose a relatively conservative control condition in order to capture the information exchange above the effect of cave geometry and similarity in bat trajectories.
- **TE and CCM identify directed coupling from rear to front bat flying in the absence of obstacles.**
  This result may be counterintuitive, but it is certainly possible, given the bats’ orientation and sensing. These bats are effective users of active sensing, wherein the acoustic signal from the rear bat may discharge forward in space and hence to the front bat. It is also worth noting that a dominant coupling direction from the rear to the front individual previously has been identified in animal group motion in a study with meerkats [21]. However, to the best of our knowledge, our study is the first that indicates the possible presence of rear-to-front coupling in animals using active sensing.
- **TE and CCM interpret coupling differently.**
  As detailed in the Materials and Methods section, TE and CCM use fundamentally different approaches: TE measures information transfer, whereas CCM identifies the causal variable. The distinction between TE and causal effect was discussed in [38]. Particularly, TE with the assumption of a traditional first-order Markov process [23] means the current position of one bat is only affected by the values of one time step prior, which may be overcome by incorporating the idea of information storage [39]. On the contrary, CCM is a tool to detect the causal variable and is not an indicator of information transfer. In other words, TE picks up the traces of significant information transfer, whereas CCM inspects the dominant influence between a pair of time series variables (i.e., coupled turning behavior between bats in this study). As a result, it is not surprising that the results differ, as we used two different tools. However, we can interpret consistency between CCM and TE results by relating the concepts. For these data, CCM infers causality when TE suggests unidirectional information exchange, e.g., with NM pairs; CCM does not indicate causal coupling when TE suggests bidirectional information exchange, e.g., all, MC, and MT pairs. Therefore, these tools capture different features of the same phenomena.
- **TE is sensitive to the choice of independent variables used.**
  To explore the sensitivity of TE results to the choice of independent variable, we adopted the multivariate functionality, whereby 3D velocity time series can be used to determine the coupling between two bats. Note that an analogous analysis was not performed for CCM since that algorithm only accepts univariate time series as input. The multivariate functionality in TE possesses a major limitation: namely, the method requires large datasets to build sufficiently dense 3D PDFs. Figure 6a–d show the sensitivity of the TE results to the choice of $k_{\mathrm{nn}}$, and Figure 6e presents the summary of the result when we fix $k_{\mathrm{nn}}$ equal to 8. From Figure 6e, bidirectional coupling is noted when all 30 pairs and MC bats are considered, whereas unidirectional coupling is observed for NM bats. Specifically, TE recognizes bat 2 to bat 1 as the dominant coupling direction for NM pairs, which is consistent when curvature is used as an independent variable. However, the evidence of interaction is lost for MT pairs, as the TE results are not significant above the control condition, as observed in Figure 6e. We conclude that TE results are sensitive to the choice of independent variables, and this leads to inconsistent results for MT pairs. In general, each choice of independent variable requires a hypothesis to test, along with a control condition. In other words, using 3D velocity as the independent variable, the underlying hypothesis is that the pairwise interactions are manifested by changing speed and/or heading direction; when using curvature, the underlying hypothesis is that the pairwise interaction is manifested by turning. In fact, changing independent variable may change the mechanism of interaction and thus the coupling direction.

Table 2 summarizes the results, which show evidence of leadership from rear bat while pairs fly in the absence of obstacles consistently between methods (TE and CCM) as well as between the independent variables used (curvature and velocity).

- **Comparison with previous work.**

In a previous paper by our group, a preliminary study was performed with NM pairs of bats, where TE was implemented and a binning approach was used for PDF estimation [30]. Unlike in the present study, an ensembled dataset was not used; instead, analysis of each pair was performed separately. In addition, a path coupling hypothesis was used, with time-delay modifications retaining Markovianity of the process. The idea of using the time delay was to take into account the response time when adjusting a bat’s trajectory based on information received from the other bat. Trends suggested a coupled relationship between the front bat and the rear bat, although statistical significance was not achieved due to the small number of pairs used in the analysis. In the present work, we extended the experimental setup by implementing obstacles in the form of a vertical maze in bats’ flight paths in order to investigate whether bats perform additional interaction for coordinating with peers. In addition, we used the JIDT toolkit to implement TE with the KSG method, which is superior in correctly estimating of PDFs compared with the classical binning approach [26]. Furthermore, we also implemented CCM, which was not explored in the earlier study, which supports the directed coupling we found with our method. To understand whether bats rely on echolocation compared with other sensing cues, a study was performed in [3] using an approach called heading delay, which is similar to cross-correlation, on leader–follower pairs. The results demonstrated significant similarities in the heading direction for a leader–follower pair when the cross-correlation delay was less than the minimum time required for communication using echolocation, thus confirming the possible presence of alternate sensing cues. However, it was not established whether the leader bats occupy the rear or frontal position, which is a question that the analysis in this study may help answer.

To conclude, in the present study, we demonstrate two model-free methods—TE and CCM—that can be used for the quantitative inference of pairwise interactions in animal groups using simultaneously recorded trajectory data. We collected the 3D trajectories of bats flying in pairs and used an additional experimental strategy: we placed obstacles in their flight path to examine the change in pairwise interaction as compared with when obstacles are absent. Although TE has been previously used to detect coupling among individuals, CCM was applied for the first time in the context of animal group motion. The main contributions of this paper are threefold. First, TE provides evidence of information exchange in a pair of individuals as manifested in the curvature values (in other words, their turning behavior). This effect is statistically significant with respect to a control condition designed to eliminate similarities in flight path due to environmental geometry. Second, both TE and CCM unanimously detect leadership from the rear bat to the front bat when the pairs fly in the absence of obstacles. A possible interpretation of leadership from the rear bat may be bats’ use of active sensing, which allows information to propagate forward in space. Finally, we show that the interpretation of interaction is dependent on the choice of independent variables, as is shown by interaction manifesting in the bats’ curvatures but not in their velocities, such as in MT pairs.

From this study, we learn that information and dynamical systems theories can be used to understand the nature of interaction in real-world biological systems. Nevertheless, this work only begins to open a wide field of research questions that can be asked about such systems and methods that can be used to answer them. The model-free methods used to capture directed coupling between individuals can further be tested using self-propelled particle models [40], where the coupling direction can be set a priori. The trajectories generated with this modeling approach can be used as a test bed for verifying the ability of these methods to correctly identify the directed coupling. Presently, our results from the two model-free methods indicate the possible presence of coupling from the rear to the front bat in the absence of obstacles. This triggers the need for a behavioral study on how obstacles impact pairwise interaction in bats. Finally, the analysis in the present study can be further extended from pairs to larger groups of individuals, and the ways in which navigational leadership is exerted over the interaction network by a single individual or a subset of the group can be studied.

## Figures and Tables

**Table 1:** Input parameters and the outputs for TE and CCM.

Method	Input	Output
TE		
	Two time series data (*X* and *Y*) PDF estimation technique: we used the KSG method for which $k_{\mathrm{nn}}$ is the parameter.	$T_{X\to Y}$ as a function of $k_{\mathrm{nn}}$ $T_{Y\to X}$ as a function of $k_{\mathrm{nn}}$
CCM		
	Two time series data (*X* and *Y*) Embedding dimension (*E*): evaluated following the Simplex Projection method [29] Constant time-delay or lag (τ): set to 1 by default Library size (*L*)	$C_{X\to Y}$ as a function of *L* $C_{Y\to X}$ as a function of *L*

**Table 2:** Results summary.

Cond	TE Using Curvature	CCM Using Curvature	TE Using 3D Velocity	Presence of Leadership
all				No
	significant $T_{1\to 2}$ significant $T_{2\to 1}$	$C_{1\to 2}=C_{2\to 1}$	significant $T_{1\to 2}$ significant $T_{2\to 1}$	
NM				Yes
	insignificant $T_{1\to 2}$ significant $T_{2\to 1}$	$C_{2\to 1}\gg C_{1\to 2}$	insignificant $T_{1\to 2}$ significant $T_{2\to 1}$	(leadership by rear bat)
MC				No
	significant $T_{1\to 2}$ significant $T_{2\to 1}$	$C_{1\to 2}=C_{2\to 1}$	significant $T_{1\to 2}$ significant $T_{2\to 1}$	
MT				No
	significant $T_{1\to 2}$ significant $T_{2\to 1}$	$C_{1\to 2}=C_{2\to 1}$	insignificant $T_{1\to 2}$ insignificant $T_{2\to 1}$	

**Figure A1:**
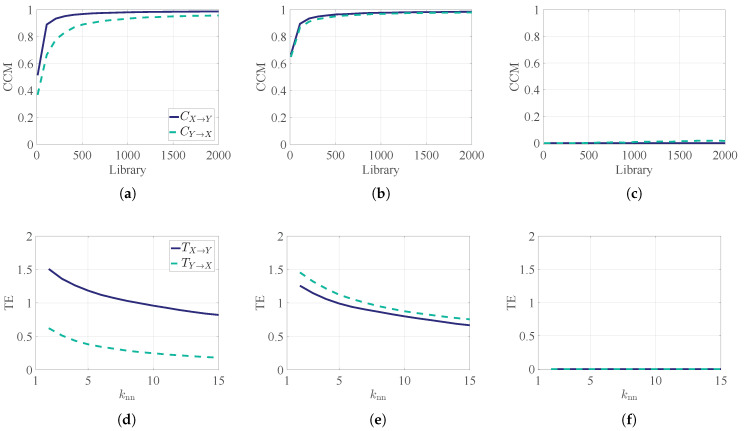
Demonstration of TE and CCM identifying three different choices of coupling. CCM is implemented on time series data from logistic equations with (**a**) unidirectional coupling (*X* driving *Y*), (**b**) bidirectional coupling, and (**c**) no coupling. TE is implemented on the same time series data with (**d**) unidirectional coupling (*X* driving *Y*), (**e**) bidirectional coupling, and (**f**) no coupling.

**Figure A2:**
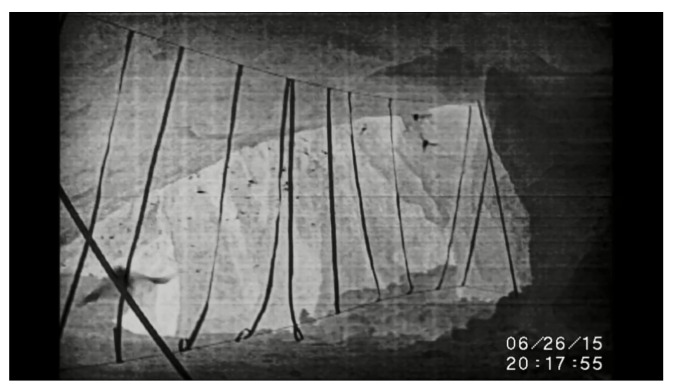
A picture of the experimental setup with obstacle course in summer 2015. The plastic tapes (5 cm wide and approximately 4.5 m long) shown as dark vertical lines were used as obstacles. The spacing between the strips was approximately 0.6 m.

**Figure A3:**
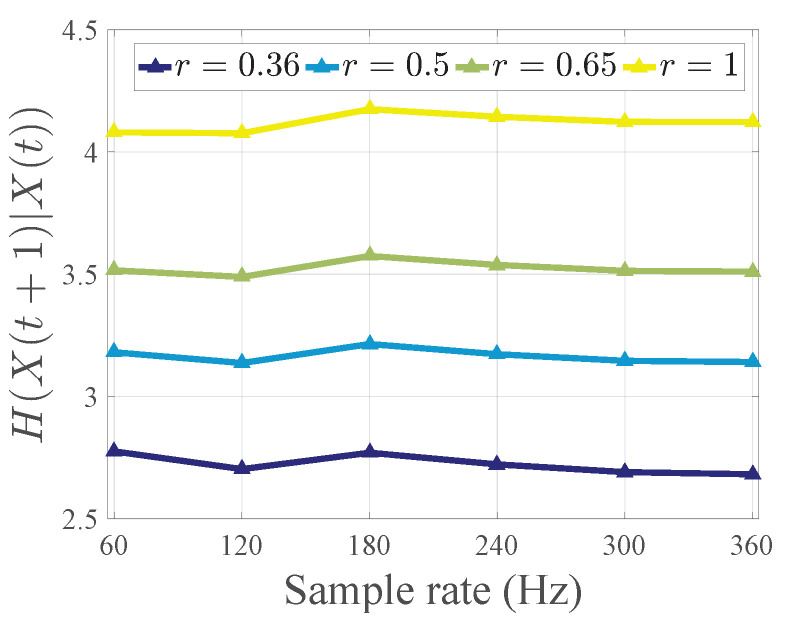
Selection of sampling rate by plotting the conditional entropy of the curvature time series of all bats. Conditional entropy as calculated for a range of sample rates for different choices of kernel widths (*r*). The location of the peak in conditional entropy was selected as the sampling rate.

**Figure A4:**
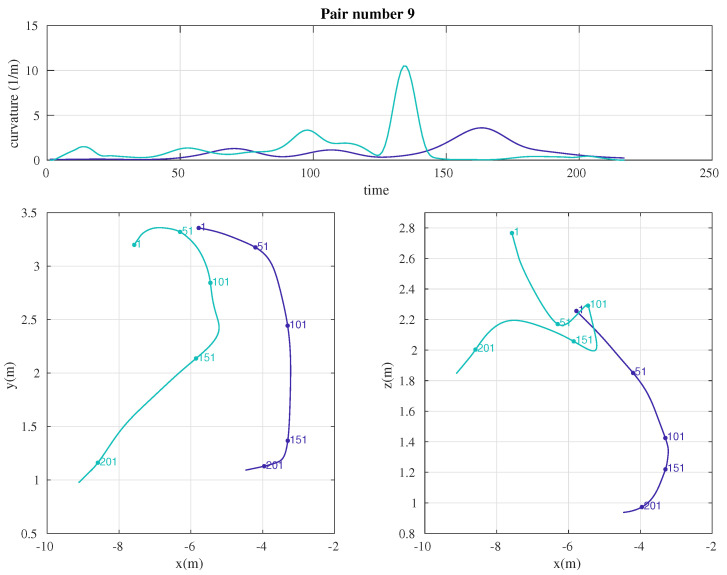
Example trajectory (in x–y and x–z planes) and 1D curvature data of an NM pair. Blue lines correspond to bat 1, and green lines correspond to bat 2. The time stamps are also provided beside the trajectory plots.

**Figure A5:**
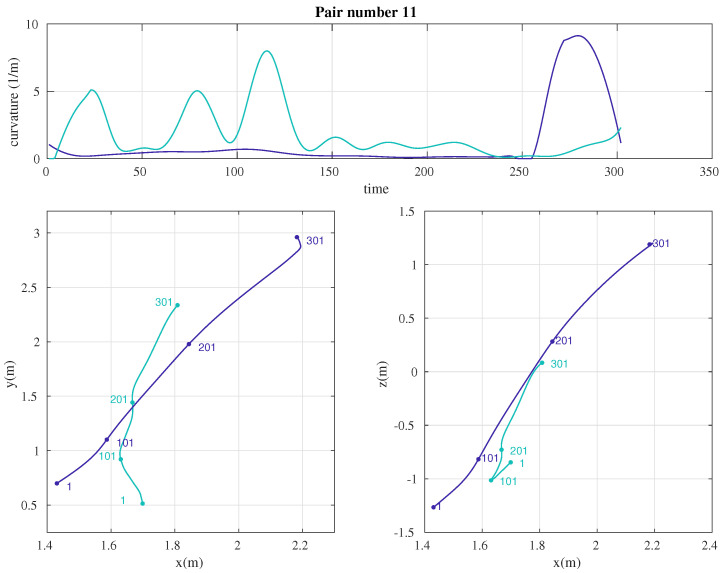
Example trajectory (in x–y and x–z planes) and 1D curvature data of an MC pair. Blue lines correspond to bat 1, and green lines correspond to bat 2. The time stamps are also provided beside the trajectory plots.

**Figure A6:**
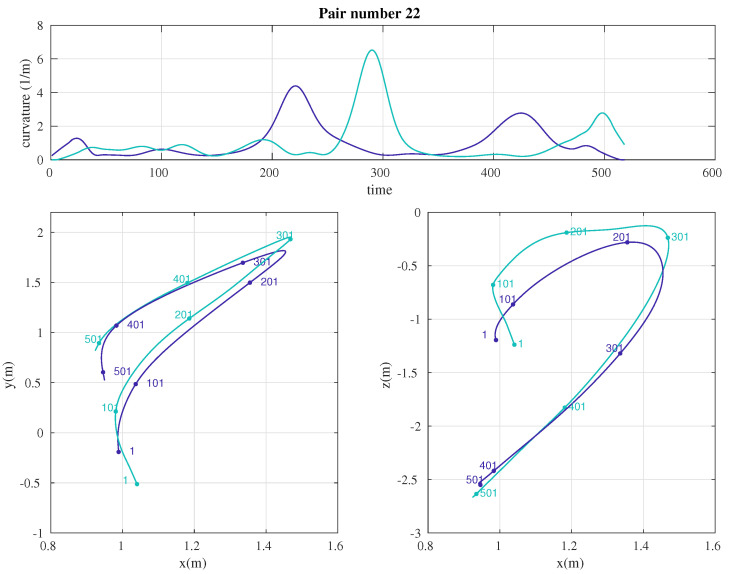
Example trajectory (in x–y and x–z planes) and 1D curvature data of an MT pair. Blue lines correspond to bat 1, and green lines correspond to bat 2. The time stamps are also provided beside the trajectory plots.

## Appendix A. Demonstration of TE and CCM

In this section, we present a brief demonstration of the different analytical tools—namely, transfer entropy, and convergent cross map—that were used in the present study to detect information exchange from time series data of bat pairs.

**Example** **1.**
*We considered a simple model system of two coupled logistic difference equations as follows:*

(A1) $$\begin{array}{ll} X(t+1) & =X(t)[3.65-3.65X(t)-\alpha Y(t)],\\ Y(t+1) & =Y(t)[3.77-3.77Y(t)-\beta X(t)], \end{array}$$

*where α and β denote the influence of Y on X, and the influence of X on Y, respectively. We chose the same initial conditions for X and Y which were set to 0.02. We further considered three scenarios: first, with bidirectional coupling, where we assigned α and β to be of same value; second, with X driving Y by choosing β>α; and third, in absence of coupling by fixing β=α=0. We applied TE and CCM in each case to examine their performance in distinguishing bidirectional from unidirectional coupling by identifying the causal variable and to verify whether they can recognize the lack of coupling.*

We chose α=β=0.5 for bidirectional causality, and α=0.15 and β=0.5 were chosen for *X* driving *Y*. Figure A1 demonstrates the results. Comparing Figure A1a,b, we observe that CCM distinguishes bidirectional causality from the unidirectional one. In the case of bidirectional causality, there is no difference in relative cross map skills, and both converge to the same value close to 1 with increasing library size. However, in the case of *X* driving *Y*, there is a difference in the convergence where $C_{X\to Y}$ converges to a higher value than $C_{Y\to X}$, which we interpret as meaning the causal variable as *X*. Transfer entropy, on the other hand, computes the information flow as a function of an input parameter to the KSG method for building the PDF, $k_{\mathrm{nn}}$. Comparing Figure A1d,e, we observe that there is a noticeable difference in the information transfer in the unidirectional coupling, as $T_{X\to Y}$ is greater than $T_{Y\to X}$ for all values of $k_{\mathrm{nn}}$, whereas, in the bidirectional case, the difference in the transfer entropy values is lost. It is important to note that causality is identified by the relative difference in TE values. On the contrary, the asymptotic value of the cross map skill in CCM is an indicator of the causal variable since these numbers are bounded. However, with real-world data being an approximation of dynamics occurring in higher dimensions, the asymptotic value of CCM is usually less than one, as in the example in [10]. Interestingly, the absence of coupling is correctly identified by both the tools, as shown in Figure A1c,f. To summarize, we verify that both these tools have the potential to capture the information exchange between time series data and can identify the causal variable; at the same time, both the tools are identified as reliable in that they do not provide false positives or false negatives.

## Appendix B. Experimental Details

**Experimental setup and tracking precision:**Figure A2 shows the experimental setup for summer 2015. The experimental setup for summer 2014 can be found in [30].

Frames from the infrared cameras used to record pairs 1–10 are 1920 × 1080 pixels, and frames from the thermal cameras used to record pairs 11–30 produce images that are 720 × 480 pixels. The reprojection errors for all images from the infrared and thermal systems are less than 0.8 and 1.2 pixels, respectively. To put this in perspective, images of tracked bats cover a minimum of 20 × 12 pixels and may be up to 44 × 24 pixels large, since bats approach the cameras as they traverse the cave. Therefore, the reprojection error is small compared with the targets we tracked for this study.

**Data processing:** Two different camera setups were used to film the videos of bats’ flights for the two summers; in particular, six GoPro cameras with infrared filters in 2014 [30] and three thermal cameras in 2015. The GoPro cameras have a frame rate of 60 Hz, whereas the thermal cameras have a frame rate of at least 25 Hz. In order to attain a consistent rate of sampling for both years’ data, we first fitted a third-order spline through three-dimensional coordinates and resampled in time at six different sample rates, ranging from 60 Hz to 360 Hz. Next, we differentiated the spline to generate the three components of velocity and acceleration data at each point. Curvature values at each point were calculated using Equation (3).

For the analysis, we concatenated the curvature time series of each bat 1 and bat 2 from all 30 pairs to build an amalgamated dataset. We selected a value for the sampling rate by plotting the conditional entropy H(X(t)|X(t−1)), as computed on the amalgamated dataset, as a function of the sampling rate. The peak in conditional entropy dictates the best choice of sampling rate [19]. Figure A3 demonstrates conditional entropy as calculated for different sample rates. To calculate conditional entropy, we used kernel estimators, and the value *r* in Figure A3 corresponds to the kernel width, which is an input parameter to evaluate the PDF. We chose a kernel estimator as it is the only available estimator that the present version of JIDT has to calculate the conditional entropy [41]. For four different choices of *r*, we identified a peak corresponding to a sample rate of 180 Hz, which we used in further analysis.

**Trajectory, curvature, and duration of flight:**Table A1 presents the trajectory lengths for bats 1 and 2, the flight duration corresponding to each pair, and their flight behavior. Note that the flight duration is the same for bat 1 and bat 2 within a given pair. This is because we disregarded the portion of the flight where only one bat was filmed and only considered the portion when both bats in a pair were filmed flying together. In Figure A4, Figure A5 and Figure A6, we present example flight paths and curvature of pairs of bats’ with three different flight behaviors: namely, NM, MC, and MT.

**Table A1:** Flight path length, duration, and behavior for all bat pairs.

Pair	Behavior	Bat 1 Path Length (m)	Bat 2 Path Length (m)	Duration (s)
1	NM	3.03	2.28	0.42
2	NM	3.26	3.95	0.68
3	NM	4.54	3.87	1.32
4	NM	2.44	2.63	0.60
5	NM	3.91	3.54	0.64
6	NM	2.07	1.86	0.41
7	NM	3.61	2.61	0.62
8	NM	2.46	1.82	0.35
9	NM	5.35	7.28	1.20
10	NM	5.87	3.46	1.05
11	MC	3.48	2.29	1.67
12	MT	20.48	39.07	13.36
13	MT	15.82	8.88	3.28
14	MC	1.71	1.36	1.03
15	MC	3.89	0.97	1.80
16	MC	6.78	8.02	2.56
17	MC	3.25	0.55	0.52
18	MC	1.89	2.85	1.36
19	MC	6.75	2.57	1.12
20	MC	0.18	0.25	0.2
21	MT	4.43	2.18	2.08
22	MT	5.15	5.72	2.87
23	MC	2.65	2.36	2.68
24	MC	4.05	2.77	1.64
25	MC	5.27	1.89	1.36
26	MT	2.94	2.95	2.6
27	MT	3.17	5.07	2.16
28	MC	3.89	1.90	0.81
29	MC	2.10	1.87	1.08
30	MC	2.79	2.45	1.32
